# Structure–Activity Relationship of Xanthones as Inhibitors of Xanthine Oxidase

**DOI:** 10.3390/molecules23020365

**Published:** 2018-02-09

**Authors:** Ling-Yun Zhou, Jia-Le Peng, Jun-Ming Wang, Yuan-Yuan Geng, Zhi-Li Zuo, Yan Hua

**Affiliations:** 1 Key Laboratory for Forest Resources Conservation and Use in the Southwest Mountains of China (Southwest Forestry University), Ministry of Education, Kunming 650224, China; zly4321@sina.com (L.-Y.Z.); dawn723@163.com (J.-M.W.); gyy18254369476@163.com (Y.-Y.G.); 2 Anhui Provincial Engineering Research Center for Polysaccharide Drugs, School of Pharmacy, Wannan Medical College, Wuhu 241002, China; 3 State Key Laboratory of Phytochemistry and Plant Resource in West China, Kunming Institute of Botany, Chinese Academy of Sciences, Kunming 650201, China; JialePeng2016@163.com

**Keywords:** xanthone, xanthine oxidase inhibitors, 3D-QSAR, gout

## Abstract

Polygala plants contain a large number of xanthones with good physiological activities. In our previous work, 18 xanthones were isolated from *Polygala crotalarioides.* Extented study of the chemical composition of the other species *Polygala sibirica* led to the separation of two new xanthones—3-hydroxy-1,2,6,7,8-pentamethoxy xanthone (**A**) and 6-*O*-β-d-glucopyranosyl-1,7-dimethoxy xanthone (**C**)—together with 14 known xanthones. Among them, some xanthones have a certain xanthine oxidase (XO) inhibitory activity. Furthemore, 14 xanthones as XO inhibitors were selected to develop three-dimensional quantitative structure–activity relationship (3D-QSAR) using comparative molecular field analysis (CoMFA) and comparative molecular similarity indices analysis (CoMSIA) models. The CoMFA model predicted a q^2^ value of 0.613 and an r^2^ value of 0.997. The best CoMSIA model predicted a q^2^ value of 0.608 and an r^2^ value of 0.997 based on a combination of steric, electrostatic, and hydrophobic effects. The analysis of the contour maps from each model provided insight into the structural requirements for the development of more active XO inhibitors.

## 1. Introduction

**Figure 1:**
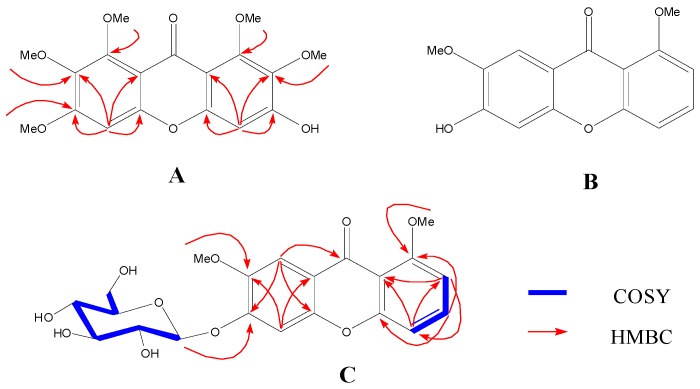
The stucture of compounds **A**–**C** from *Polygala sibirica.*

**Figure 2:**
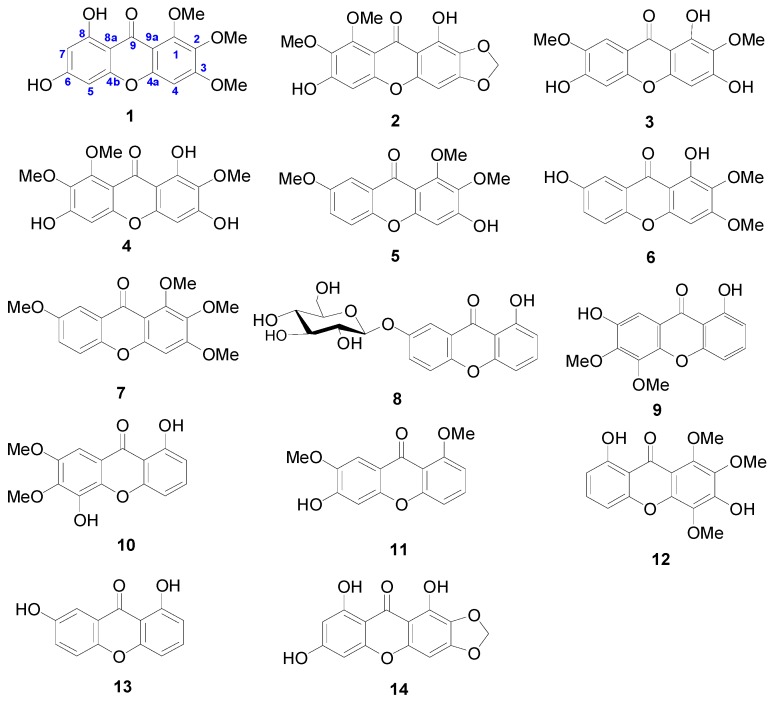
The stucture of the 14 xanthones studied.

Xanthones are a class of natural products commonly occurring in a few higher plant families [1] and have been shown to display a wide range of pharmacological properties [2,3]. An important natural source of these compounds is the Polygalaceae family which includes about 22 genera and 1300 species [4]. In our previous works [5,6], 18 xanthones were isolated from *Polygala crotalarioides*, some of which have the inhibitory activitiy against XO. This bioactivity attracted us to investigate more xanthones from other genus of this family. In the present work, 2 new xanthones (compounds **A** and **C**, Figure 1) and 14 known xanthones were isolated from *Polygala sibirica* L. var. megalopha Fr. The bioactivity results showed that all 14 tested xanthones (Figure 2) had a certain inhibition activity against XO. Today, the ligand-based drug design (LBDD) is an important part in computer-aided drug design (CADD) [7,8]. Comparative molecular field analysis (CoMFA) [9] and comparative molecular similarity indices analysis (CoMSIA) [10,11] represent the successful and widely used LBDD approaches for guiding the strategies of chemical derivative modifications [12]. Conventional CoMFA uses electrostatic and steric molecular interaction fields, while CoMSIA includes hydrogen bonding and hydrophobic fields additionally [13,14]. The alignment of molecules was important for QSAR modeling. Especially, the alignment based on docking was normally more reasonable than on common structures as it takes the binding pose in the receptor into consideration. It also provides us convenience for docking as many crystal structures of XO have been released [15,16,17,18]. Herein, we studied the structure–activity relationship (SAR) of xanthones as inhibitors of XO through 3D-QSAR techniques based on molecular docking. We expect that our work could provide complementary and useful information to discover novel potent XO inhibitors.

## 2. Results and Discussion

### 2.1. Identification of New Compounds

Compound **A** was obtained as a yellow amorphous powder and its molecular formula (C_18_H_18_O_8_) was determined based on the HR-ESI-MS data (*m*/*z* 575.1667 [M + Na]^+^ calcd. for 575.1741). The UV spectrum revealed maxima at 208, 245, 277, and 308 nm. The IR exhibited absorption bands at ν_max_ 3425 (O–H), 1611 (chelated C=O), 1510 (aromatic ring) cm^−1^. Its ^13^C-NMR spectral data (Table 1) showed the presence of five methoxyl signals, 1 carbonylcarbon signal, as well as 12 aromatic carbons. These spectral data indicated the presence of a hexasubstituted xanthone. The carbon signal at δ_C_ 172.8 is characteristic for a doubly unchelated carbonyl [19] and four of five methoxyl signals (56.6, 60.9, 61.0, 61.7, and 61.8) were typical of di-ortho-substituted methoxyl groups (δ_C_ > 60) [20]. The ^1^H-NMR spectrum of **A** showed two aromatic proton singlets at δ_H_ 6.63 and 6.87, assignable to H-4 and H-5, respectively. The structure of **A** was further confirmed by the HMBC spectrum (Figure 1). On the basis of the above evidence, the structure of **A** was elucidated as 3-hydroxy-1,2,6,7,8-pentamethoxyxanthone.

Compound **B** was isolated as an amorphous yellow powder. Based on the ESI-MS of **B** indicating [M + Na]^+^ at *m*/*z* 311, its molecular formula was deduced to be C_15_H_12_O_5_. The UV spectrum showed absorption maxima at 205, 245, 285, and 358 nm, suggesting the presence of a xanthone skeleton. The ^1^H-NMR spectrum of **B** showed the presence of three methoxyl signals at 3.84 and 3.86, two singlets of aromatic protons at 6.84 (s, H-5) and 7.39 (s, H-8) and three mutual coupling signals of aromatic protons. Finally, compound **B** was determined as 6-hydroxy-l,7-dimethoxyxanthone by the comparison of the literature [21].

Compound **C**, amorphous yellow powder，was analyzed for C_21_H_22_O_10_ by HR-ESI-MS at *m*/*z* 457.1110 [M + Na]^+^ (calcd. for 457.1105). The ^1^H-NMR and ^13^C-NMR spectra of **C** (Table 2) were similar to those of **B**, except that signals corresponding to the sugar moiety included an anomeric proton signal (δ = 5.88 (1H, d, *J* = 7.5 Hz)) and anomeric carbon signal (δ = 101.6). On acid hydrolysis, **C** gave a d-glucose unit by comparison with the authentic sample on GC. The relative configuration of the glucose residue was deduced to be β by the coupling constants (*J*) of 7.5 Hz. In the HMBC spectrum, the glucosyl group was proposed at C-6 by the correlations from Glc-H-1 (δ_H_ 5.88, *J* = 7.5 Hz) to C-6 (δc 153.2). Furthermore, the structure of compound **C** was further confirmed by the HMBC and ^1^H-^1^H COSY spectrum. Therefore, the structure of **C** was formulated as 6-*O*-β-d-glucopyranosyl-1,7-dimethoxyxanthone.

### 2.2. CoMFA and CoMSIA Analysis

**Figure 3:**
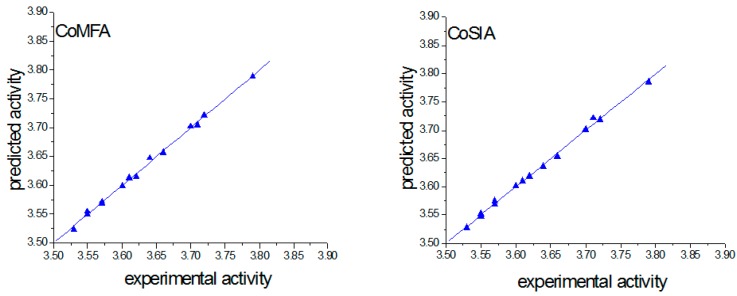
Plot of experimental against predicted pIC_50_ by CoMFA and CoMSIA.

Table 3 showed the predicted pIC_50_ values for 14 xanthones (Figure 2), and the differences between the predicted and experimental pIC_50_ values are given as well. The CoMFA model was obtained using the training set of 14 xanthones, and all corresponding statistical parameters were listed in Table 4. Figure 3 showed the relationship between the CoMFA 

Predicted and experimental pIC_50_ values, from which it can be seen that all points are located near the diagonal line. The high q^2^, r^2^ and *F*-values, along with the low SEE value, indicated a good statistical correlation and reasonable predictability of the CoMFA model. The best CoMSIA model was developed using three descriptor fields: steric, electrostatic and hydrophobic fields. According to the COMFA analysis method, a good statistical correlation, and reasonable predictability of the CoMSIA model were also indicated from Table 4 and Figure 3. 

### 2.3. Verification of Model Reliability

Using the established model, we predicted the relative values of all 14 xanthones and sorted their values. From the order of the original activity, the prediction of the model and the order of docking scoring (Table 5), it was found that the order of activity of Compounds **4**, **8**, and **14** with relatively high activity was relatively in front, and compound **13** with the ranking of the lowest active was still backward, and the order of the predicted value was basically the same as that of the original activity. The results showed that the activity value predicted by the model is reliable.

### 2.4. CoMFA and CoMSIA Contour Maps

**Figure 4:**
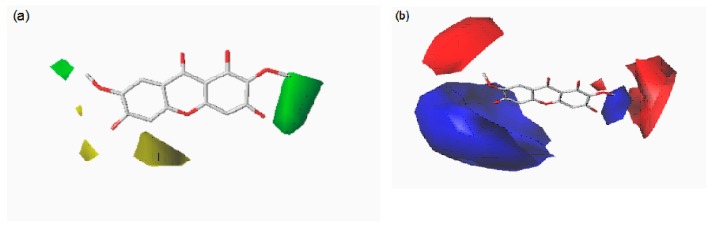
Contour maps of CoMFA: steric (**a**) and electrostatic (**b**) based on Compound **3**.

**Figure 5:**
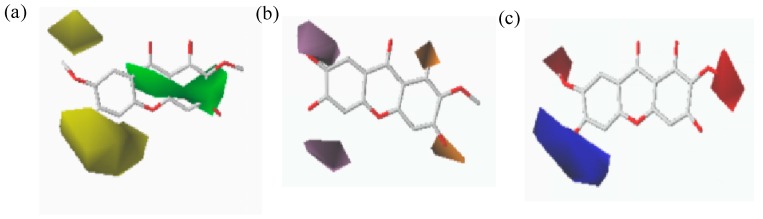
Contour maps of CoMSIA: steric (**a**); electrostatic (**b**); and hydrophobic (**c**) based on Compound **3**.

Contour maps of CoMFA and CoMSIA (Figure 4 and Figure 5) gave a semi-quantitative information for the structure of all 14 xanthones. Compound **3** was used as the reference molecule. The green contours represent regions where bulky substituents would increase the activity, while the yellow contours represent regions where the steric bulky group would be unfavorable. Furthermore, the blue and red contours depict the position where positively charged groups and negatively charged groups would be favorable, respectively. In addition, the purple and orange regions represent the effect of the electrostatic field on the activity of the compounds. The introduction of hydrophobic groups in the purple region can improve the activity of the compound, and the introduction of the hydrophilic group in the orange region is beneficial to the activity.

Compound **3** has the highest activity value, and its various maps are representative. However, because the molecular docking is not as regular as the skeleton overlap, the situation of each molecule placed into the active cavity docking is different. In order to analyze comprehensively, each molecule would be placed in the three-dimensional field, electrostatic field, and hydrophobic field.

**Figure 6:**
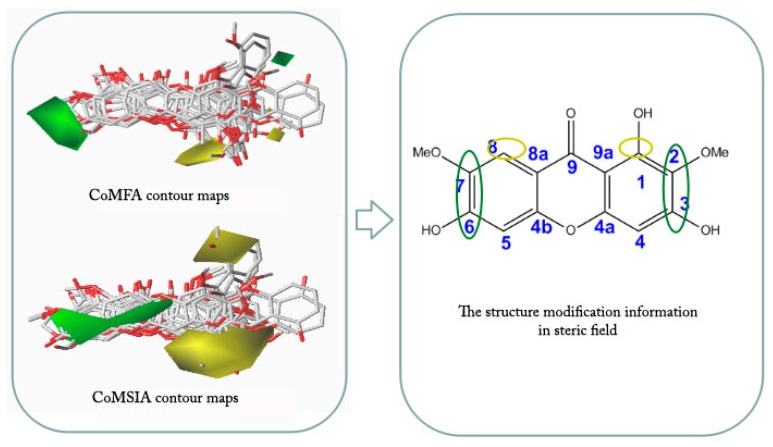
Steric field views of 14 xanthones.

As shown in Figure 6, each molecule was placed in a steric field and analyzed in detail. It was found that the yellow region tended to be close to the 1, 8 position of the compound, indicating that the two positions should be not suitable for introduction of groups. This may be the reason why Compound **3** with no substitutions at 8-position was more potent than Compound **4** with a methoxy substituent. The green region tended to approach the 2-, 3-, 6-, and 7-position of the compound, indicating that these positions may be suitably introduced into the group and that the larger groups may favor the activity increase, which explain why Compound **13** without the glycosyl group at 7-position had weak activity compared to Compound **8** with a glycosyl substitution. The 4,5-position information of the compound was not obvious. 

**Figure 7:**
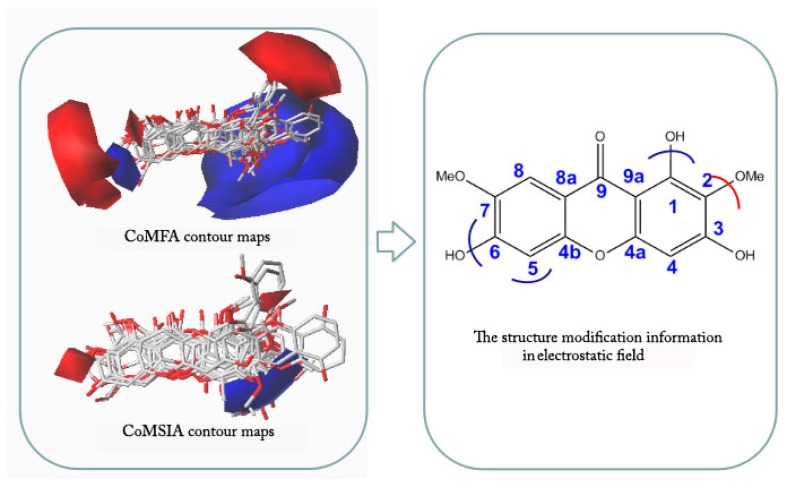
Electrostatic field views of 14 xanthones.

As shown in Figure 7, each molecule was placed in an electrostatic field and analyzed in detail. It was found that the blue region tended to be close to the 1, 8 position of the compound, indicating that the introduction of positively charged groups at this site should increase the activity. Compound **3** with no substituent at the 8-position had better activity compared to Compound **4** being oxygen-substituted. This also confirmed the previous inference that its 1- and 8-position should be no substituent. The red region tended to be close to the 7-position of the compound, indicating that the introduction of a negatively charged group can increase the activity of the compound.

**Figure 8:**
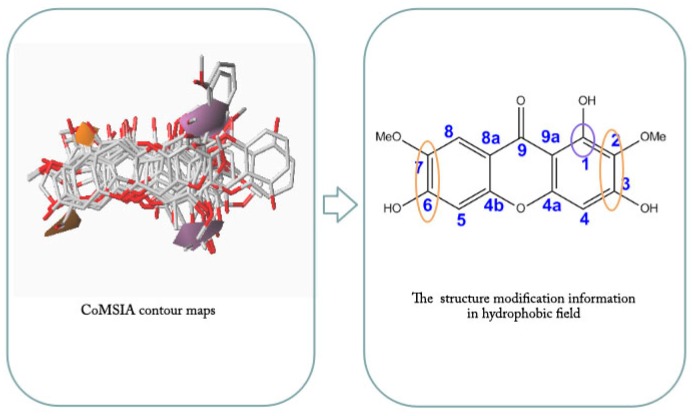
Hydrophobic field views of 14 xanthones.

The incorporation of a hydrophilic group at the 2, 3, 6, 7 position of the compound (as shown in the orange region of Figure 8) should enhance the activity while the purple region tended to be close to 1, 4, indicating the introduction of hydrophobicity at both positions groups were conducive to increasing the activity.

## 3. Experimental Section

### 3.1. General Information

Optical rotations were recorded using a P-1020 digital polarimeter (Jasco, Tokyo, Japan). The UV spectra were measured on a UV-2401PC spectrophotometer (Shimadzu, Suzhou, China). The IR spectra were recorded as KBr pellets on a Tensor-27 spectrometer (Bruker, Bremen, Germany). The NMR spectra were recorded on an AM-400 spectrometer (Bruker) with TMS as an internal standard. ESI-MS and HR-EI-MS-TOF-MS were measured on a Bruker HTC/Esquire spectrometer and a Bruker Daltonics Flex spectrometer, respectively. GC analysis was carried out on an HP-5890 II system (Gentech Scientific, Alto, CA, USA) equipped with a FID detector and a HP-20M capillary column (25 m × 0.32 mm × 0.3 μm). Column chromatography was performed with silica gel (Qingdao Marine Chemical Industry Factory, Qingdao, China) and Sephadex LH-20 (GE Healthcare Bio-Sciences AB, Fairfield, CT, USA) and reversed-phase C18 silica gel (40–60 μm, Merck, Darmstadt, Germany). TLC was performed with silica gel GF254 (Qingdao Marine Chemical Industry Factory). Fractions were monitored by TLC and spots were visualized by heating after spraying with 5% H_2_SO_4_ in ethanol.

### 3.2. Plant Material

The whole plant of *P. Sibirica* L. Var megalopha Fr. were collected in Yongshan, Yunnan Province, People’s Republic of China. A voucher specimen (HUA002) was identified by Prof. Fan Du (Southwest Forestry University) and was deposited at the College of Forestry, Southwest Forestry University.

### 3.3. Extraction, Isolation, and Characterization

The 75% ethanol extract of the whole dry plant of *Polygala sibirica* (5.0 kg) was purified using chromatography with D101 macroporous resin and the column contents were eluted with H_2_O, 35% EtOH, 65% EtOH, and 95% EtOH, using a gradient. The 65% EtOH elute was concentrated in vacuo to afford a residue (480 g), which was subjected to silica gel column chromatography, using a CHCl_3_–MeOH gradient (50:1→0:1) as the eluent to separate it into 13 fractions (Fr1–Fr13). Fr2 (8 g) was applied to MCI-gel column with MeOH–H_2_O gradient (1:1→1:0) to afford four fractions (Fr2A-Fr2D). Fr2B (1.9 g) was chromatographed on a silica gel column and eluted in a step gradient manner with a CHCl_3_:MeOH (15:1→0:1) gradient system to afford five fractions 2B1-2B5. Subfraction 2B2 (0.9 g) was further purified by Sephadex LH-20 (CHCl_3_:MeOH = 1:1) chromatography and semi-prepared HPLC (CH_3_CN:H_2_O = 40:60, λ = 254 nm) to produce compound **A** (retention time = 11.5 min, 5.6 mg) and compound **B** (retention time = 8.2 min, 10.4 mg). Subfraction 2B5 (0.5 g) was further purified by Sephadex LH-20 (CHCl_3_:MeOH = 1:1) chromatography and semi-prepared HPLC (CH_3_CN:H_2_O = 30:70, λ = 254 nm, retention time = 14.1 min) to produce compound **C** (8.6 mg). 3-hydroxy-1,2,6,7,8-pentamethoxyxanthone (**A**): yellow amorphous powder; HR-ESI-MS *m*/*z*: 385.0899 [M + Na]^+^ (calcd. for 385.0894, C_18_H_18_O_8_Na^+^); ${\left[\alpha \right]}_{D}^{20}$ −22.59° (*c* 0.9, MeOH); UV (CH_3_OH) λ_max_ (logε): 308 (4.29), 277 (4.07), 245 (4.62), 208 (4.46); IR (KBr) *ν*_max_: 3425, 2936, 2853, 1611, 1510, 1468, 1424, 1384, 1273, 1123, 1080, 1061, 993 cm^‒1^; ^1^H-NMR and ^13^C-NMR data see Table 1.

*6-Hydroxy-l,7-Dimethoxyxanthone* (**B**): yellow amorphous powder; ESI-MS *m*/*z*: 317 [M + K]^+^, 311 [M + Na]^+^ and 289 [M + H]^+^; UV (CH_3_OH) λ_max_ (logε): 358 (4.022), 285 (3.987), 245 (4.186), 205 (4.517)； ^1^H-NMR (400 MHz, DMSO-*d*_6_) *δ*: 7.62 (1H, t, *J* = 8.5 Hz, H-3), 7.39 (1H, s, H-8), 7.04 (1H, d, *J* = 7.8 Hz, H-4), 6.90 (1H, d, *J* = 8.2 Hz, H-2), 6.84 (1H, s, H-5), 3.86 (3H, s, OMe-1), 3.84 (3H, s, OMe-7); ^13^C-NMR (100 MHz, DMSO-*d*_6_) δ: 160.0 (s, C-l), 106.1 (d, C-2), 134.4 (d, C-3), 109.5 (d, C-4), 102.3 (d, C-5), 153.8 (s, C-6), 146.0 (s, C-7), 105.6 (d, C-8), 173.6 (s, C-9), 157.4 (s, C-4a), 114.4 (s, C-8a), 111.3 (s, C-9a), 150.5 (s, C-l0a), 56.1 (q, OMe-1), 56.8 (q, OMe-7).

*6-O-β-d-Glucopyranosyl-1,7-Dimethoxyxanthone* (**C**): yellow amorphous powder; HR-ESI-MS *m*/*z*: 457.1110 [M + Na]^+^ (calcd. for 457.1105, C_21_H_22_O_10_Na^+^); ${\left[\alpha \right]}_{D}^{20}$ −112.78° (*c* 0.8, MeOH); UV (CH_3_OH) λ_max_ (logε): 360 (4.137), 283 (4.215), 247 (4.586), 203 (4.717); IR (KBr) *ν*_max_: 3417, 2925, 1620, 1503, 1476, 1433, 1385, 1270, 1177, 1077, 806, 583 cm^‒1^; ^1^H-NMR and ^13^C-NMR data, see Table 2.

### 3.4. Acid Hydrolysis of Compound ***C***

A solution of **C** (3 mg) in 1 M HCl (3 mL) was heated in a water bath at 70 °C for 6 h. After cooling, the reaction mixture was neutralized with NaHCO_3_ and extracted with CHCl_3_. The aqueous solution was further concentrated to dryness and dissolved in 1 mL pyridine. Then hydroxyl-ammonium hydrochloride (1 mg) was added. The solution were kept in a 80 °C water bath for 30 min, followed by treatment with 1 mL Ac_2_O and kept in a 90 °C water bath for another 40 min. The authentic monosaccharide samples were treated similarly as the hydrolysis products. Finally, 1 μL of these acetylated sugars were injected into a HP-20M capillary column using N_2_ as carrier (oven temp. 210 °C) to be analyzed by GC using a FID detector (detector temp. 280 °C). Ultimately, the sugar was determined to be d-glucose by comparing the retention time with that of standard sample: *t*_R_: d-glucose 8.686 min.

### 3.5. Bioassay of Xanthine Oxidase Inhibitory Activity

The enzyme xanthine oxidase catalyzes the oxidation of xanthine to uric acid. Inhibition of xanthine oxidase results in a decreased production of uric acid. The uric acid production was measured according to the increasing absorbance at 290 nm. Test solutions were prepared by adding xanthine (final concentrations 50 μM), hydroxylamine (final concen-tration 0.2 mM), EDTA (final concentration 0.1 mM), and flavonol glycosides in four concentrations (2.5, 5.0, 10.0, 20.0 μM). The reaction was started by adding 0.2 mL of xanthine oxidase (6.25 mU/mL) in a phosphate buffer solution (pH = 7.50, 200 mM). The mixture (total 1 mL) was incubated for 30 min at 37 °C. Prior to the measurement of uric acid prodution, the reaction was stopped by adding 0.1 mL of HCl (0.58 M) [22]. The IC_50_ values were calculated by the mean data values from three determinations.

### 3.6. Dataset Preparation

All 14 xanthones and their biological activities were performed by our own. Two-dimensional structures of xanthones were sketched by Chem-Draw 12.0 software and then converted into three-dimensional structures by GOLD software (Version 3.1, Genetic Optimization of Ligand Docking, London, UK). Correctly re-docking in the protein crystal structure is a key requirement to predict the binding poses. Since the XO protein (PDB entry: 1N5X) was downloaded from the protein data bank [1N5X], it was prepared by removing water and solvent molecules, adding hydrogen atoms, loading charges, and amending amino acid residues. The original molecule TEI-6720 in the protein was extracted as a reference ligand in the docking process to explore the root mean square deviation (RMSD). As a result, the scoring function of *CHEMPLP* and the docking template of chemscore kinase were selected as this combination afforded a RMSD of 0.506. Therefore, these 14 compounds were docked by the selected docking template and scoring function to obtain the poses which were employed in the QSAR modeling later. Then the activities of all xanthones along with its pIC_50_ values were transformed and were given in Table 3. The values of pIC_50_ of the dataset set ranging from 3.53 to 3.79 are supposed to contribute to the appropriateness of the training set xanthones. 

### 3.7. CoMFA and CoMSIA Studies

The 3D-QSAR modelling was carried out in Sybyl-X 2.0 (Tripos, Inc., St. Louis, MO, USA). The CoMFA models were developed on the training set of 14 molecules and validated by the test set of the same molecules, in which Lennard-Jones and Coulomb potentials were employed to calculate the CoMFA steric and electrostatic fields respectively, using the sp^3^ carbon as the probe atom having a + 1 charge to determine the magnitude of the field values. The steric and electrostatic energy cutoff was set to 33 kcal/mol with the column-filtering value set to 2.0 kcal/mol. The CoMSIA was similar to CoMFA about the fields around the aligned molecules, and the other descriptor named hydrophobic effect was calculated together.

### 3.8. Validation of 3D-QSAR Models

Internal validation was performed using partial least square (PLS) analysis on the training set, in which leave-one-out (LOO) method was used to determine the cross-validated q^2^ and the optimal number of components (ONC), and non-cross-validation was performed to calculate conventional r^2^ using the same number of components. The external validation ($r_{\mathrm{pred}}^{2}$) was recommended for the assessment of the predictive abilities of CoMFA and CoMSIA models on the test set. Predictive $r_{\mathrm{pred}}^{2}$ were calculated as

(1) $$r_{\mathrm{pred}}^{2}=1-\mathrm{PRESS}/\mathrm{SD}$$

in which SD is the sum of squared deviations between the experimental inhibitory activity of the test set and the mean experimental inhibitory activity of the training set molecules, PRESS is the sum of squared deviations between the predicted and experimental activity values for every molecule in the test set.

## 4. Conclusions

The chemical constituents of *Polygala sibirica* led to two new xanthones. Combined with the previously isolated xanthones from the *Polygala crotalarioides*, we investigated the activity of xanthones against XO. On the basis of these data, three-dimensional quantitative structure–activity relationships (3D-QSAR) models using CoMFA and CoMSIA were utilized to investigate structural requirements for improving potency of xathone derivatives as XO inhibitors. Authentic CoMFA model (q^2^ = 0.613, r^2^ = 0.997) and CoMSIA model (q^2^ = 0.608, r^2^ = 0.997) were developed and validated. Analysis of model parameters and contour maps provided details of the structure–activity relationship. We expect this is quite helpful to develop new xathone XO inhibitory lead compounds, which can provide a promising approach to treat gout.

## Figures and Tables

**Table 1:** The ^1^H (600 MHz) and ^13^C-NMR (125 MHz) data of **A** in DMSO-*d*_6_.

Position	δ_H_	δ_C_
1		153.2 s
2		138.6 s
3		156.6 s
4	6.63 (s)	98.8 d
5	6.87 (s)	96.2 d
6		157.7 s
7		138.9 s
8		152.8 s
9		172.8 s
4a		152.8 s
8a		110.4 s
9a		109.6 s
10a		152.9 s
OMe-1	3.80 (s)	61.7 q
OMe-2	3.74 (s)	61.0 q
OMe-6	3.89 (s)	56.5 q
OMe-7	3.72 (s)	60.9 q
OMe-8	3.81 (s)	61.8 q

**Table 2:** The ^1^H (600 MHz) and ^13^C-NMR (125 MHz) data of **C** in DMSO-*d*_5_.

Position	δ_H_	δ_C_
1		160.8 s
2	6.97 (d, 8.2)	110.0 d
3	7.51 (t, 8.2)	134.4 d
4	6.76 (d, 8.2)	105.9 d
5	7.59 (s)	103.8 d
6		153.2 s
7		147.3 s
8	7.89 (s)	106.6 d
9		174.7 s
4a		158.3 s
8a		117.2 s
9a		112.5 s
10a		150.8 s
OMe-1	3.83 (s)	56.1 q
OMe-7	3.67 (s)	55.7 q
Glc-1	5.88 (d, 7.5)	101.6 d
2	4.42 (m)	74.5 d
3	4.44 (m)	78.3 d
4	4.40 (m)	70.9 d
5	4.35 (m)	79.2 d
6	4.57 (d, 11.7) 4.40 (m)	62.2 t

**Table 3:** Xanthones and their observed and predicted (pre.) XO inhibition activities.

Number	pIC_50_	CoMFA		CoMSIA	
		pre.pIC_50_	Residue	pre.pIC_50_	Residue
1	3.66	3.657	−0.003	3.654	−0.006
2	3.64	3.648	0.008	3.636	−0.004
3	3.79	3.789	−0.001	3.785	−0.005
4	3.71	3.705	−0.005	3.721	0.011
5	3.55	3.551	0.001	3.547	−0.003
6	3.60	3.599	−0.001	3.601	0.001
7	3.62	3.615	−0.005	3.619	−0.001
8	3.72	3.722	0.002	3.719	−0.001
9	3.57	3.569	−0.001	3.57	0
10	3.57	3.572	0.002	3.575	0.005
11	3.55	3.555	0.005	3.553	0.003
12	3.61	3.613	0.003	3.61	0
13	3.53	3.524	−0.006	3.528	−0.002
14	3.70	3.702	0.002	3.701	0.001

**Table 4:** Statistical parameters of CoMFA and CoMSIA model.

Method	CoMFA	CoMSIA
*PLS statistics*		
*q* ^2^	0.613	0.608
*SEP*	0.065	0.066
Optimum components	6	6
*r* ^2^	0.997	0.997
*SEE*	0.005	0.006
*F*-ratio	426	0.997
*Field distribution*		
Steric	0.632	0.152
Electrostatic	0.368	0.316
Hydrophobic		0.532

**Table 5:** The relative experimental and predicted (pre.) values order of 14 xanthones.

Number	pIC_50_	coMFA-pre. pIC_50_	coMSIA-pre. pIC_50_	Original Activity Order	CoMFA pre. Activity Order	CoMSIA pre. Activity Order	Docking Score	Docking Score Order
1	3.66	3.657	3.654	3	8	4	72.23	2
2	3.64	3.648	3.636	8	4	8	71.41	8
3	3.79	3.789	3.785	4	14	14	60.48	9
4	3.71	3.705	3.721	14	2	2	60.59	14
5	3.55	3.551	3.547	1	3	3	53.7	4
6	3.6	3.599	3.601	2	7	7	54.48	3
7	3.62	3.615	3.619	7	12	12	45.98	6
8	3.72	3.722	3.719	12	6	6	64.35	10
9	3.57	3.569	3.570	6	10	1	62.93	5
10	3.57	3.572	3.575	9	9	10	54.32	1
11	3.55	3.555	3.553	10	11	9	50.12	11
12	3.61	3.613	3.610	5	5	11	49.86	12
13	3.53	3.524	3.528	11	1	5	38.95	7
14	3.7	3.702	3.701	13	13	13	61.05	13
